# High-resolution ethograms, accelerometer recordings, and behavioral time series of Japanese quail

**DOI:** 10.1038/s41597-023-02820-w

**Published:** 2024-01-02

**Authors:** Catalina Simian, Florencia Belén Rossi, Raul Hector Marin, Lucas Barberis, Jackelyn Melissa Kembro

**Affiliations:** 1 Laboratorio de Biología Reproductiva y Evolución, Instituto de Diversidad y Ecología Animal (IDEA, UNC-CONICET), Córdoba, Argentina; 2 Instituto de Investigaciones Biológicas y Tecnológicas (IIByT, UNC-CONICET), Córdoba, Argentina; 3 Universidad Nacional de Córdoba (UNC), Facultad de Ciencias Exactas, Físicas y Naturales, Instituto de Ciencia y Tecnología de los Alimentos (ICTA), Córdoba, Argentina; 4 Universidad Nacional de Córdoba, Facultad de Ciencias Exactas, Físicas y Naturales, Cátedra de Química Biológica, Córdoba, Argentina; 5 Universidad Nacional de Córdoba, Facultad de Matemática, Astronomía Física y Computación, Córdoba, Argentina; 6 Instituto de Física Enrique Gaviola (IFEG, UNC-CONICET), Córdoba, Argentina

**Keywords:** Animal behaviour, Data publication and archiving

## Abstract

Although many small vertebrates are capable of performing high-speed behaviors, most studies continue to focus on low-resolution temporal scales (>>1 s). Herein, we present video-recordings, behavior time series, and the computer software for video-analysis of Japanese quail within social groups. Home-boxes were monitored using both top and side video-cameras. High-resolution ethograms were developed for analyses. Pairs of females were assigned as either controls or using one of two methods for attachment of an accelerometer (patch or backpack). Behavior was recorded during 1 h on the first 2-days, sampled at 1 s intervals (days 1 and 2). On day 8, an unfamiliar male was placed in the home-box and its behavior was recorded during the first 10 min, sampled every 1/15 s. Male accelerometer recordings were also obtained. Video-recordings and resulting detailed high-resolution behavioral time series are valuable for reuse in comparative studies regarding the temporal dynamics of behavior within social environments. In addition, they are necessary for the assessment of novel machine learning algorithms that could be used for deciphering the output of accelerometer recordings.

## Background & Summary

Evaluating individual behavioral dynamics within social groups is technically challenging. Not only does it require monitoring the behavior of each animal continuously over time, but also, the resolution of obtained data must be sufficient to capture social interactions between conspecifics. These social interactions can potentially be performed at high-speeds. For example, male mice have been shown to change their trajectory and/or acceleration in less than 150 ms in response to female vocalizations^1^. If the surrounding environment is enriched and complex, and/or is large, this adds further challenges in obtaining the needed high quality data. Different methodological approaches have been developed to face these challenges. For instance, video-analysis software, such as idTracker, can track individuals within a group by automatic identification of unmarked animals^2,3^. Alcala *et al*.^4^ implemented this technology in small groups of Japanese quail housed in home-boxes, and showed that an aggressive dominant conspecific can decrease the behavioral complexity of subordinates through synchronization of locomotor activities^4^. However, although valuable, such tracking software is basically limited to studies of locomotor dynamics or spatial use, but does not provide information regarding other behaviors. Remote sensors, such as accelerometers, have been proposed as a method to obtain behavioral information even in large complex environmental settings^5^. Accelerometers measure the acceleration associated with movement and body position of the animal. When acceleration is measured in three dimensions (x, y, and z), three values corresponding to each dimension are recorded at every sampling point, resulting in acceleration vectors a_x_, a_y_, and a_z_, respectively. Previous studies have shown that it is feasible to use body-mounted accelerometers for jump detection by hens in different non-cage housing configurations^6^. However, the implementation of this type of technology is not straightforward. It involves attaching an accelerometer to the animal, and afterwards classifying the data from the accelerometer using specific algorithms such as neural networks k-nearest neighbor^7–9^, in order to obtain the resulting behavioral time series. Hence, implementing remote sensors to evaluate behavioral dynamics requires prior pilot studies deemed at assessing the best attachment method as well as the best algorithm for processing.

Herein, we present time series data sets obtained from Japanese quail within social groups. All behavioral data was collected at either real time or at fixed sampling intervals of ≤1 s, depending on the objective of each test. Data sets are part of a larger study aimed at assessing whether the method for attaching the accelerometer to the animal’s body could induce behavioral changes, whether birds adapt to the devices over time and if accelerometer recordings can be used to detect male reproductive behavior. High resolution data, with sampling interval <100 ms, was only considered necessary for evaluating the male-females interaction test since social interactions can potentially occur at high speeds. Prior experimentation that found that locomotor (spatial displacement) events in Japanese quail can be as short as 100 ms^10^. Thus, given this reported possible high-speed transition between behaviors, accelerometer data was obtained at 25 Hz (i.e. 25 data points per second) in males, and effort was made to analyze the corresponding video-recording at the highest resolution possible in our setup (15 data points per seconds). The high-resolution ethograms developed for data recording are presented herein as well as the code of a customized MATLAB app^11^ designed to obtain high-resolution behavioral data from the original video files^12^.

**Fig. 1:**
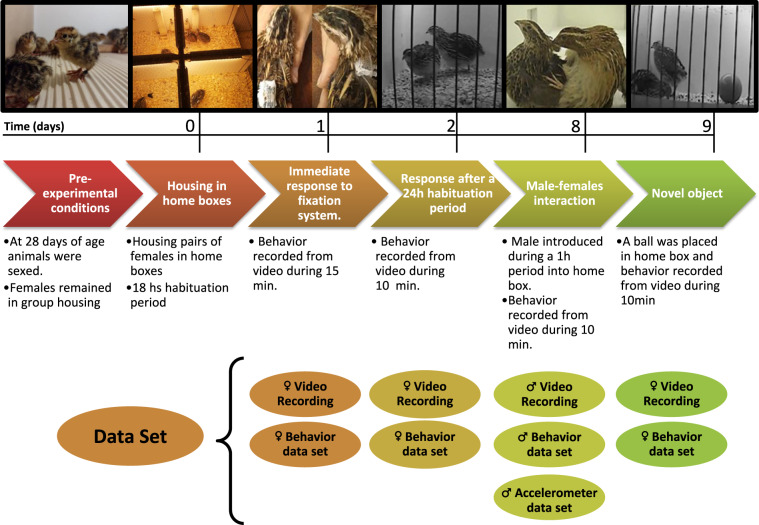
Experimental timeline. Photographs represent from left to right: Birds hatching. Pair housing of females in home boxes. The two methods for attaching the accelerometer to the bird, the Patch and the Backpack systems. Side camera recording of the immediate and 24 h behavioral response to devices. Example of male reproductive behavior. Side camera recording of the novel object test. The timeline shown below the photographs is expressed in days. Day 0 is the moment females were housed in home boxes. The title of the corresponding test and/or experimental step is shown in filled colored boxes and the summary of experimental details are indicated in white boxes below each title. The resulting video recordings and datasets for each test are depicted in colored circles bellow the test. ♀ and ♂ indicate time series obtained in females and males, respectively.

**Fig. 2:**
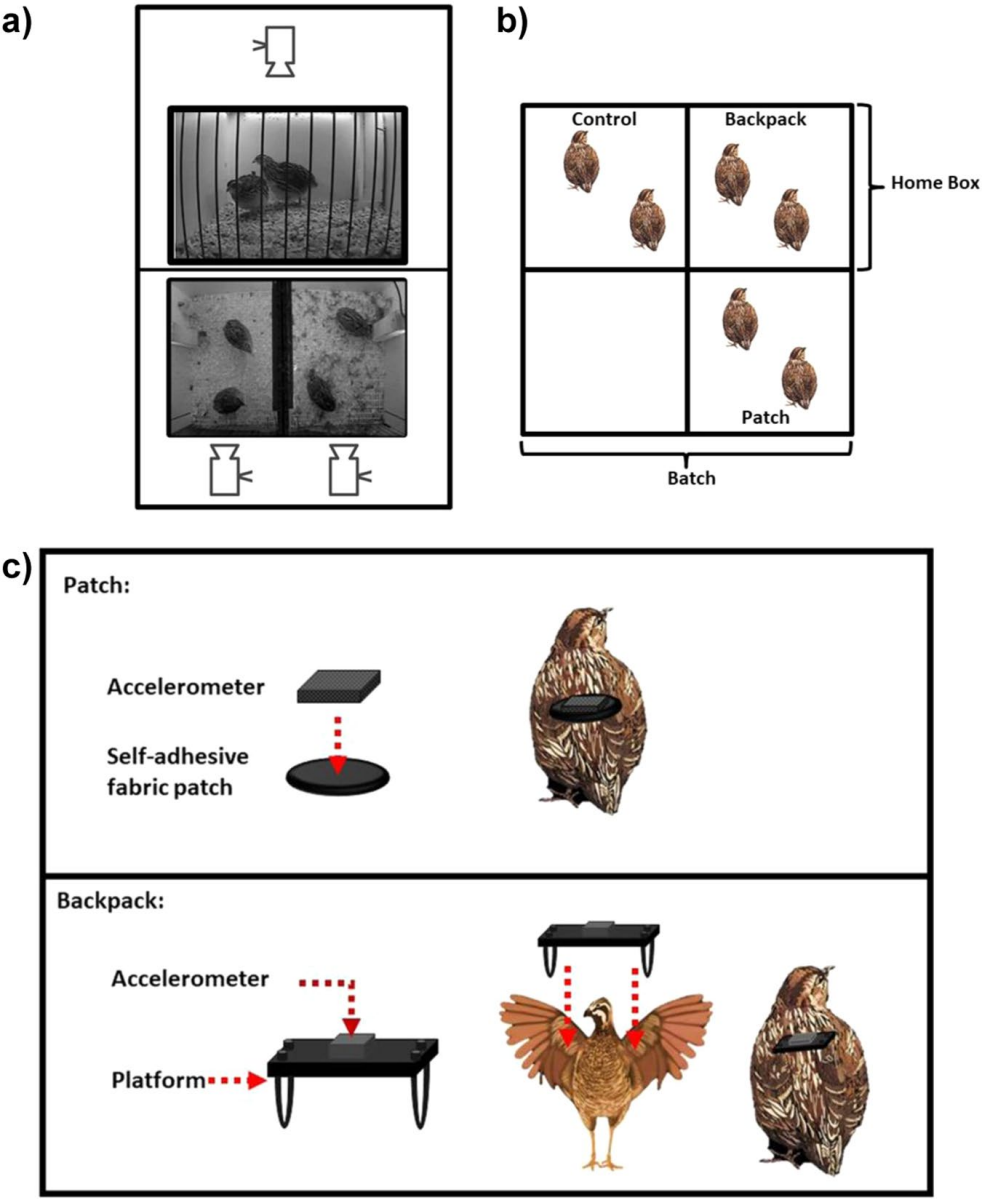
Video-recording and experimental setup. (**a**) Cameras were positioned above the box as well as on the other side of a wire mesh wall providing side and top views of each box. (**b**) Schematic representation of an experimental batch. The relative position of each treatment group was changed in each batch to avoid confounding effects of the box position. (**c**) Representation of the patch and backpack methods are shown.

The experimental timeline is presented in Fig. 1. Adult females were housed in pairs in home-boxes and monitored with a recording system that included a top and side camera connected to a computer (Fig. 2a,b). Two different systems for attaching the accelerometers were assessed (Fig. 2c). The first consists of an accelerometer attached to a patch made of fabric glued to the synsacrum area of the bird. The second, an accelerometer attached to a backpack (i.e. plastic platform fitted by 2 elastic fabric bands around the wings’ base). A control group with birds handled similarly but without an accelerometer attached was also evaluated. Female behavioral time series were recorded at 1 s sampling intervals during a 15 min period (900 data points per bird per day) immediately after placement of the device (i.e. immediate response test; Fig. 1). Behavior was also assed at real time for a 1 h period 24 h after attaching accelerometers (Fig. 1). On day 8, the male-females interaction test was performed (Fig. 1) where a male under the same treatment condition (control, patch or backpack) was introduced during a 1 h period into home-boxes. All observable male behaviors were recorded at a 1/15 s resolution during the first 10-min (9000 data points per male). In males, accelerometer data was also collected during the total 60-min of testing at 1/25 s intervals. Lastly, on day 9, a novel object test was performed. Accelerometer recordings were not obtained for females in any of the tests.

These data sets have already been analyzed in three different contexts. First, as mentioned previously, to evaluate the effects of attaching the accelerometer to adult quail on behavior dynamics, as well as, the potential habituation process^13^. Second, in the assessment of the probability distribution of the duration of adult male quail behavioral events within social environments^10^. Three, to begin training neural networks to detect male reproductive behavior from accelerometer recordings^14,15^. Data from all tests are provided as data time series, thus are flexible for reuse since variables can be estimated from this raw data, e.g. percent of time performing a behavior, transitions probabilities between behaviors, behavioral event number and durations. This is extremely valuable for assessment of probability distribution of behavioral variables in different experimental contexts and for studies focused on the temporal dynamics of behavior. Moreover, given the high-resolution of the data obtained in the male-female interaction test the data sets could be valuable for reuse in comparative studies, between different species and contexts, regarding the high-speed temporal dynamics of behavior within social environments. Also, time series can be reused for training and comparing different algorithms to automatically detect diverse behaviors from accelerometer recordings. Video recordings of all tests are available thus can be reanalyzed using different criteria for recording behavior.

## Methods

The experiment was conducted with quail (*Coturnix japonica*) according to the “Guide for the Care and Use of Laboratory Animals”^16^. Protocol was approved by the Institutional Animal Care and Use Committee (CICUAL) of the Institute for Biological and Technological Research (IIByT, UNC-CONICET), n°6.

### Animal husbandry

Eggs were collected for 10 consecutive days from adult quails from the Institute’s breeding stock. These eggs were stored in a refrigerator at 15 °C until incubation. For 17 days, the eggs were placed in an incubator/hatcher with automatic egg rotation (turning the eggs by approximately 45° every 1 h), temperature and humidity controls. During the first 14 days of incubation the eggs were rotated, the average temperature was maintained at 37.8 °C and the relative humidity at 65%. In the last 3 days of incubation, the eggs were transferred to trays without rotation, the temperature was reduced to 37.5 °C and the relative humidity was set at 62%. Thus, the micro-environmental conditions inside the incubator were kept optimal for embryo development.

One hundred and eighty chicks were housed in the rearing room distributed in 6 white melamine rearing boxes measuring 90 × 80 × 60 cm (width, length, height respectively). The boxes included an automatic temperature control system that was set at 37.5 °C for the first week and then decreasing by 3.0 °C per week until room temperature (24 to 27 °C) was reached in the fourth week. Throughout the entire study quails were subjected to a daily 14:10 h light:dark cycle (300 to 320 lx), lights on at 6 am.

At 28 days of age, the animals were sexed according to plumage coloration^17^ and wing banded with a designated identification (ID) number. At 50 days of age, 40 females remained in two rearing boxes while 20 males were housed individually in 40 × 20 × 25 cm home cages (length × width × height). The remaining animals were used for other studies. Food and water provision continued *ad libitum* throughout the entire study.

Wing-band ID numbers were used to randomly assign birds in pairs of females (with a corresponding male), to an experimental group (Control, Backpack and Patch) and to batches. A total of 18 pairs of females and 18 males were studied. Three home boxes, one from each experimental group, were evaluated simultaneously (i.e. batch) during the 9-day experimental period (Figs. 1, 2). The assignment of the given experimental group to a box position varied between batches. Within each batch birds were not acoustically isolated from each other.

On day 0 (Fig. 1) pairs of adult females of at least 70 days of age were housed in white-melamine board home boxes (Fig. 2) measuring 40 × 40 × 40 × 40 cm (length × height × width), while males remained in their home cages. In home boxes wood shavings covered the floor, and had an automatic waterer and a feeder. One of the walls of the box was made of wire bars, and 20 cm behind it a side camera was placed. A second camera was also suspended 1 m above the boxes. Both cameras were connected by closed circuit to a computer. Females remained undisturbed for at least an 18 h habituation period prior to initiation of experimental period on day 1. All tests were performed starting at approximately 3 h after the light onset (i.e. 9 am). Daily routine maintenance was performed after testing, and was minimal during the 9-day experimental period, since it only involved removing visible eggs. Automatic drinkers, large tolva feeders and wood shavings flooring did not need maintenance during the experimental period.

### Experimental treatment groups

On day 1, each pair of females and the corresponding experimental male (see section Male-females interaction test) were handled similarly by two experimenters, one held the bird while the other plucked the feathers from the synsacrum area, elevated wings, and, when corresponding, placed the accelerometers using one of two attachment systems (Fig. 2c):

#### BACKPACK

In this method, a plastic platform was used to hold the accelerometer. The platform has two elastic bands on their sides that are passed around the base of the quail’s wings (based on the model proposed by Pellegrini *et al*. 2019) as shown in Fig. 2c. Hence, the accelerometer remains on the dorsum above the scapula area. Similar backpacks that hold sensors have been previously studied in chicken, *Gallus gallus domesticus*^18,19^.

#### PATCH

In this method, the accelerometer was attached to a self-adhesive fabric patch (“Athletic Tape”), subsequently surgical glue was used to adhere the device to the synsacrum area of the animal’s dorsum, which was previously plucked manually, as shown in Fig. 2c.

For both attachment systems a TechnoSmArt@ accelerometers (9.5 × 15 × 4mm 0.7 g) were used. Accelerometer recordings from females were not obtained in this study. Control birds remained without wearing any accelerometers. After placement of devices females and males were returned to their home boxes and home cages, respectively.

It is important to note that due to technical issues, including hard drive failure, a few videos from each test were lost, as well as half of the male accelerometer recordings, hence final sample size number varied in each test (see individual tests and section data records).

### Behavioral studies

#### High-resolution ethogram

Tables 1–6 show the high-resolution ethogram obtained from frame-by-frame behavioral observations performed prior to the study in individually housed birds and birds within social groups.

**Table 1:** Behaviors associated with preening and plumage maintenance.

BEHAVIOR	PHOTO	DESCRIPTION
Preening		Uses beak to lift and accommodate feathers of the wings, abdomen, or hindquarters.
Upright preening		Uses short beak movements to lift feathers near the neck while standing in an upright position. Usually alternating with vigilance.
Sitting shaking		Shakes and partially erect feathers while resting the abdomen on the ground.
Standing Shaking		Short display of a large part of the plumage partially erect while shaking the body in a standing position.
Wing stabilization		Rapidly unfurls of a wing or wing movement to stabilize position
Scratching		Uses a claws to scratch abdomen
Dust Bath		Bird performs raking movements with the bill, scratching or scraping movements with the legs, tossing dust into the air with the wings and undulating the body under the dust shower, rubbing the head and body in the dust, and vigorous feather ruffling and shaking^26^.

**Table 2:** Behaviors associated with foraging which do not involve active displacement.

BEHAVIOR	PHOTO	DESCRIPTION
Ground pecking		Uses the beak to touch the ground substrate while standing
Feeding		Uses the beak to ingest feed particles. Usually from a feeder trough.

**Table 3:** Behaviors that do not involve spatial displacement.

BEHAVIOR	PHOTO	DESCRIPTION
Vigilant while sitting		Vigilant in an upright body position while their legs are kept flexed on the ground.
Vigilant		Bird stands upright, with the neck and body stretched, the head elevated above the shoulders, and eyes open.
Standing		Stands in an upright position with their two feet on the ground. Neck and body are not stretched.
Resting		Bird lies on the abdomen with their legs flexed under the body
Sleeping		Birds lie on the ground with their legs stretched towards the side and eyes closed.

**Table 4:** Behaviors associated with spatial displacement.

BEHAVIOR	PHOTO	DESCRIPTION
Walking		Performing steps in any direction (speed is slower than running).
Walking squatting		Walking with their legs flexed.
Exploring		Moving the head towards the ground but without touching the ground with its beak.
Running		Accelerated walking, body is elongated parallel to the ground.
Jumping		The bird elevates on the air separating vigorously both feet from the ground. Usually is not moving their wings.

**Table 5:** Behaviors associated with interaction of individuals with objects.

BEHAVIOR	PHOTO	DESCRIPTION
Pecking each other’s accelerometer		A bird uses its beak to touch the partner´s body area where an accelerometer is positioned.
Pecking their own accelerometer		During preening intervals the bird turns the head and pecks at its body area where an accelerometer is positioned.
Pecking ball		Uses the beak to touch a ball introduced in the testing box.

**Table 6:** Social interactions between conspecifics.

BEHAVIOR	PHOTO	DESCRIPTION
Pecks		When one bird raises its head and vigorously pecks the other bird’s body (usually on the head).
Non-aggressive social interaction		The bird brings its head towards its partner’s head.
Grabs		Male catches (“grabs”) with their beak the female´ neck or head region feathers.
Mounts		While performing a grab, the male approaches a female from behind or a side, and places both feet on the dorsal surface of its torso, stepping over the females’ body.
Cloacal contacts		During mounting, the male lifts his tail and tilts his pelvis underneath the other bird and briefly presses its cloaca against the female.

#### Immediate response

After handling and/or attaching accelerometers, the birds were returned to their home box and their behavior was videotaped for 15 minutes. Twenty-two behaviors were recorded according to the high-resolution ethogram (Tables 1–6). A MATLAB app, developed in MATLAB R2018a, based on the Behaviour Collect program^20^, was used for the recording (see section Code Availability for details). This application allows recording the observed behaviors using alphanumeric keys during the specified sampling interval. To facilitate data annotation, after the video recording of the sampling interval is displayed, a pause allows the observer to record the behavior performed by the animal using the corresponding alphanumeric key. Upon pressing the alphanumeric key, the video recording of the next sampling interval is shown. In this test a sampling interval of 1 s was used, resulting in a time series of 900 behavioral data (1 data per second for 15 minutes) per animal. Fifteen video-recordings of pairs of females are available (Table 7) from 5 control, 6 backpack and 4 patch experimental groups.

**Table 7:** Overview of the file names uploaded to figshare grouped in file sets according to the test performed.

Test		File name	Description
Immediate response		Inmediate_response_Batch#_Box#_Female#_Treatment	Behavioral time series of two females sampled at 1 s intervals during the first 15 min after handling and/or attaching devices (N = 15 pairs of females).
Response after a 24 h habituation period		Response_24h_Batch#_Box#_Treatment	Behavioral time series recorded by continuous observation of two females during 10 min using ANY-maze, 24 h after handling and/or attaching devices (N = 15 pairs of females).
Novel Object		Novel_object_Batch# _Box#_Female#_Treatment	Behavioral time series recorded by continuous observation using ANY-maze of both females, during the first 10 min after placing a novel object within the home-box (N = 14 pairs of females).
Male-females interaction	Behavioral recordings	Male_behavior_Batch#_Box#_Treatment	Behavioral time series of the male sampled at 1/15 s intervals during the first 10 min after being placed into the home-box (N = 13 males).
	Accelerometer recordings	Male_accelerometer_ Batch#_Box#_Treatment	Accelerometer recordings of the male sampled at 1/25 s intervals during the first hour after being placed into the home-box. (N = 6 males).

N indicates the total number of files within each test.

#### Response after a 24 h habituation period

On day 2, cameras were activated remotely and female behavior was recorded, during 10 min, 24 h after handling and/or attaching devices. To facilitate data collection, the ANY-maze Video Tracking System was used to analyze video-recordings, which also allows recording the behaviors detected by the observer by means of an alphabetical key. This program does not use a fixed sampling interval, but rather records the time in which the corresponding key is pressed. Also, based on the results from day 1, only the most frequently observed behaviors were studied. Namely, preening, shaking, pecking the ground, feeding, vigilant, standing, resting, sleeping, walking, exploring, running, pecking each other’s accelerometer and pecking their own accelerometer (see definitions Tables 1–6). Fifteen video-recordings of pairs of females are available (Table 7) from 4 control, 6 backpack and 5 patch experimental groups.

#### Male-females interaction test

Females remained undisturbed during a week, taking into consideration the similar study performed by Pellegrini *et al*., that showed that this period is sufficient for habituation to a backpack type device^21^. On the 8th day of experimentation (Fig. 1), a male of the same treatment group (see section Experimental treatment groups) was randomly assigned to a home-box (i.e. control, patch or backpack). Before being introduced into the home-box of the corresponding pair of females, a protocol to synchronize the video with the accelerometer signal was performed. With the accelerometer and the video recorder turned on, each male was gently handled to favor a position in which the male stayed relatively still for 1 minute in order to favor a period of minimal acceleration. After this, the experimenter gently performed a series of up and down movements with the hand that held the bird, generating a characteristic acceleration signal important for later synchronization. These movements were followed by another minute of stillness. If the male presented a period of unrest that could hinder the synchronization process the clock the process restarted. After this synchronization protocol, the male was released and remained in the home-box during a 1 h period. In the resulting video, every frame (15 frames per second) was analyzed during 10 min, thus a 67 ms (1/15 s) sampling interval was used. In each frame the behavior being performed by the male was registered by an experimenter (Tables 1–6) using the alphanumeric key assigned with a MATLAB app developed specifically for this purpose (see Code Availability section). Thus, from each of these trials, two different time series were obtained, one consisting of the series of behaviors performed during that time and the other consisting of the acceleration vectors. Six accelerometer time series are available and 13 video recordings from 4 controls, 4 backpack and 5 patch experimental groups (Table 7).

#### Novel object test

On day 9 of the experiment (Fig. 1), a 5 cm diameter plastic blue ball was placed in the center of each home box. After the placement of the ball, behavior was recorded for the next 10 min onto a computer. At the end of this period, the ball was removed from the box.

**Fig. 3:**
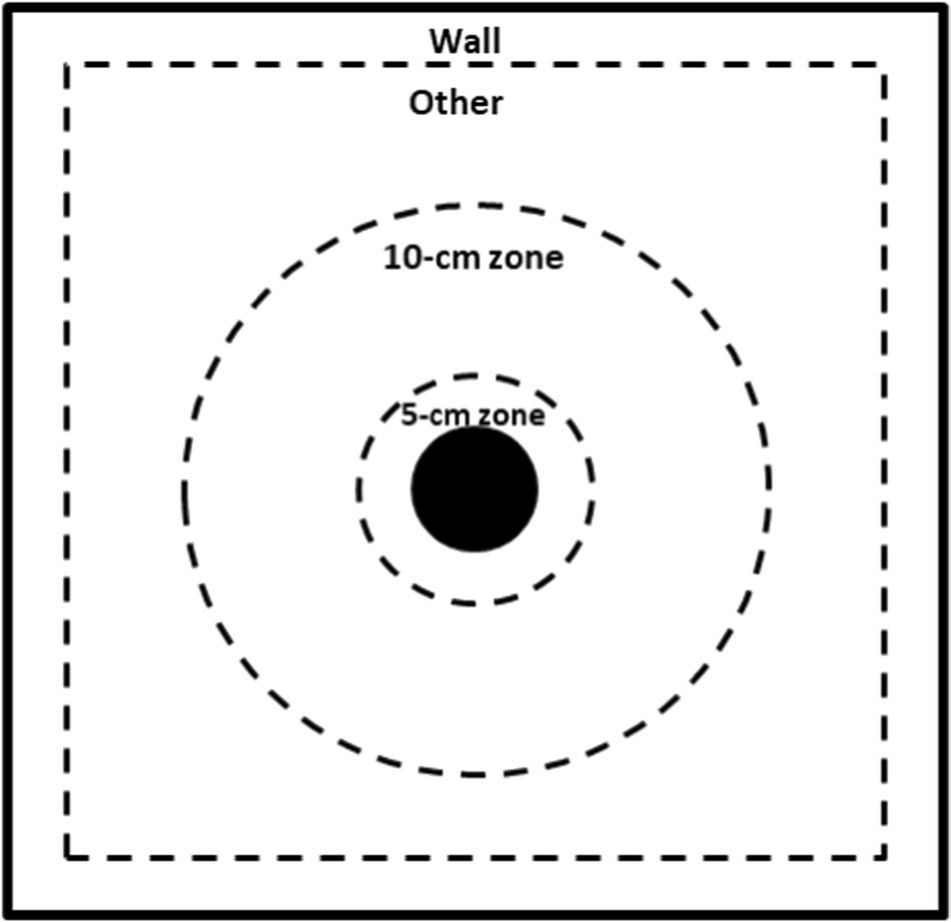
Schematic representation of the 4 zones of the home box evaluated in the novel object test. The outer square (solid black line) denotes the wall of the home box, while the black filled circle in the middle indicates the ball used. Two consecutive circles (discontinues black lines) represent a radius of 5 and 10 cm around the ball (i.e. 5-cm and 10-cm zones, respectively). A third zone line (discontinues black lines) marks a 10 cm region where animals are physically located against the walls of the box (i.e. wall zone). The area between the wall and the 10-cm zone is considered as other.

For the analysis of this test from video recordings using the ANY-maze Video Tracking System, the box was delimited in imaginary zones where each of the individuals could be found with respect to the location of the ball. In ANY-maze areas were drawn for visual references as markers to facilitate recordings. The female was considered to be in a given area if most of her body was located within it (Fig. 3):

- 5-cm zone: area contained within a 5-cm radius around the ball.
- 10 cm zone: area contained in a radius of 10 cm around the ball.
- Wall: if animals were physically located against the walls of the box.
- Other: area contained between the 10 cm zone and the Wall zone.

It should be noted that in this test, proximity and physical interaction with the ball is considered to represent a reduced fear response to the novel object^22^. The duration of “freezing” behavior (animal remaining immobile) was estimated as the time from the initiation of the test to the first step the animal took in any direction.

ANY-maze was also used to record behaviors associated with the interaction with the novel object, the accelerometer or their conspecific. As stated previously, this program does not use a fixed sampling interval, but rather records the time in which the corresponding key is pressed. Specifically, pecking at the ball, jumping near the box wall, shaking, pecking at conspecific, pecking each other’s accelerometer and pecking their own accelerometer (Tables 1–6). Fourteen video-recordings of pairs of females are available, corresponding to five controls, five backpack and four patch experimental groups (Table 7).

### Variable estimation from behavioral time series

**Fig. 4:**
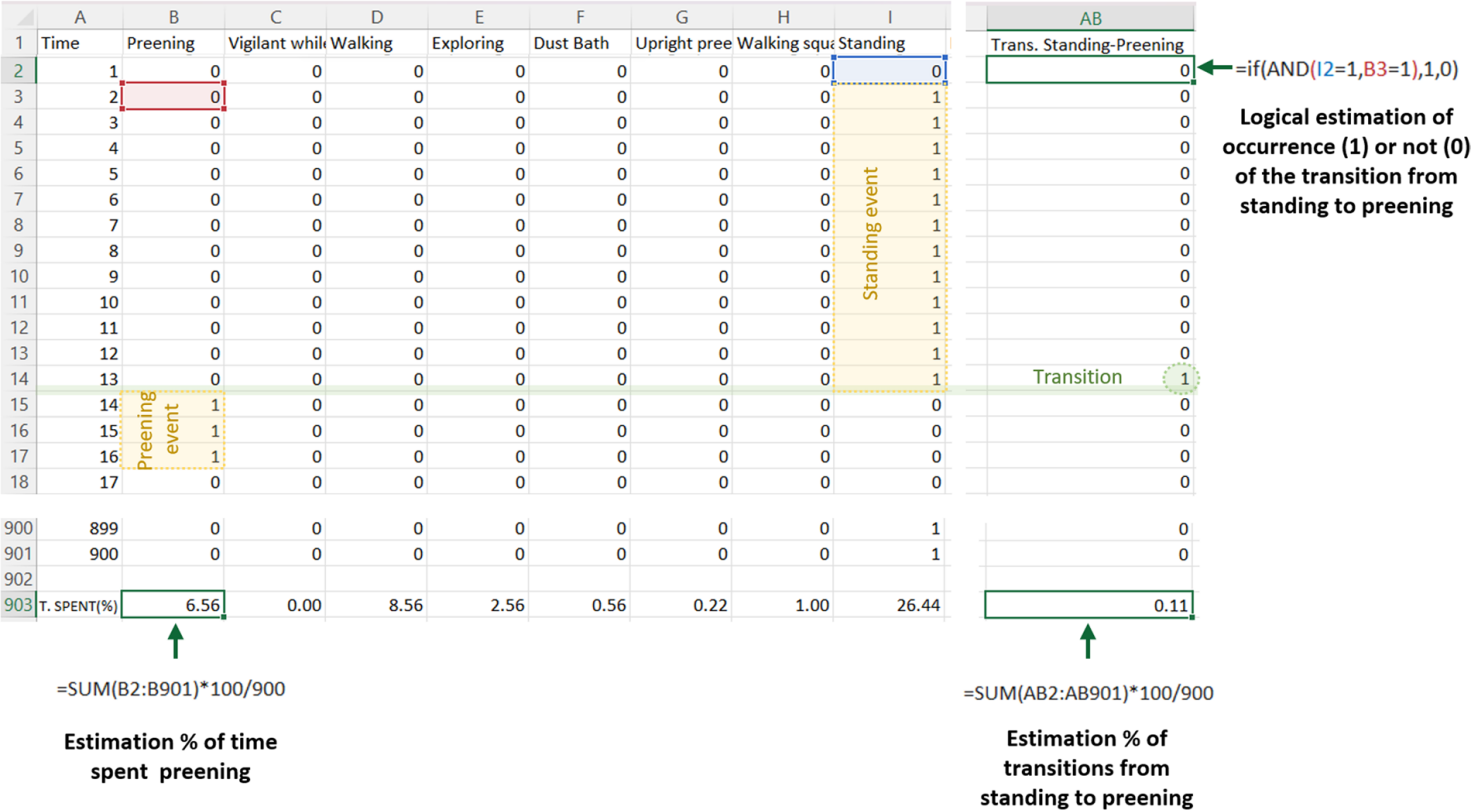
Examples of different variables that can be calculated from data time series. Data from file “Immediate_response__Batch6_Box 3_Female1_Backpack.csv” was imported in Excel. The sheet shows the behavioral time series (columns) obtained in the immediate response test for one of the females from the backpack experimental group. Note that within each column mutually exclusive binary values are observed; a one represents that the behavior is being performed, while a zero indicates that it is not being performed in the time interval. Straightforward estimation of percent of time spent performing a behavior is shown as an excel formula. Event duration can be estimated as the continuous time performing a behavior, thus for the two examples, the standing event has a duration of 12 s and the preening event of 3 s, as marked with a yellow box. A transition between standing and preening events is highlighted in green. In order to detect transitions in Excel a separate column can be generated using a logical formula that states that if the bird is standing at a specific time interval and preening in the next time interval than the transition occurred and a 1 is recorded, if not a zero is recorded (i.e. the value 1 within the green circle denotes the occurrence of a transition standing to preening). At the end of the AB column the percent of standing to preening transitions is estimated using an excel formula.

From all behavioral datasets straightforward estimations of time spent performing a given behavior, number and duration of behavioral events (i.e. continuous time spent performing a given behavior^10^) and transitions between behaviors can be estimated from each animal (Fig. 4). To facilitate variable estimation from data, each behavior is presented in a separate column, with each row indicating the sampling interval (i.e. time point, Fig. 4). A binary 1-0 annotation is used to indicate whether or not the behavior is performed. Thus, the number 1 is recorded for each interval in which the behavior was observed in the column corresponding to a particular behavior and a 0 is recorded when it is not observed. Since the behaviors defined are mutually exclusive each row can only present a single column containing the value 1 (Fig. 4).

Variable estimation from data sets is straightforward and does not require specialized software. An example in Excel is provided in Fig. 4. For time series in which the sampling interval is constant (i.e. immediate response and male-females interactions), percent of time performing a given behavior is obtained by adding all values recoded in the column, dividing it by the number of observations, and then multiplying this result by 100 (Fig. 4). Transitions between behaviors (Fig. 4) can be estimated by calculating the probability that at each time point (t) the behavior (x_t_) is followed by the behavior y (y_t+1_). Behavior events can be calculated in each column by estimated the continuous amount of time the animal is performing a given behavior (in other words the number of continuous values of 1; Fig. 4).

For the tests without constant sampling intervals (i.e. response after a 24 h habituation period and novel object test) the time interval (first column) must be taken into consideration in estimations. For this task, publicly available code for transforming data into equally spaced constant sampling intervals is available^23^.

### Comparisons between videos, observational data and accelerometer recordings

**Fig. 5:**
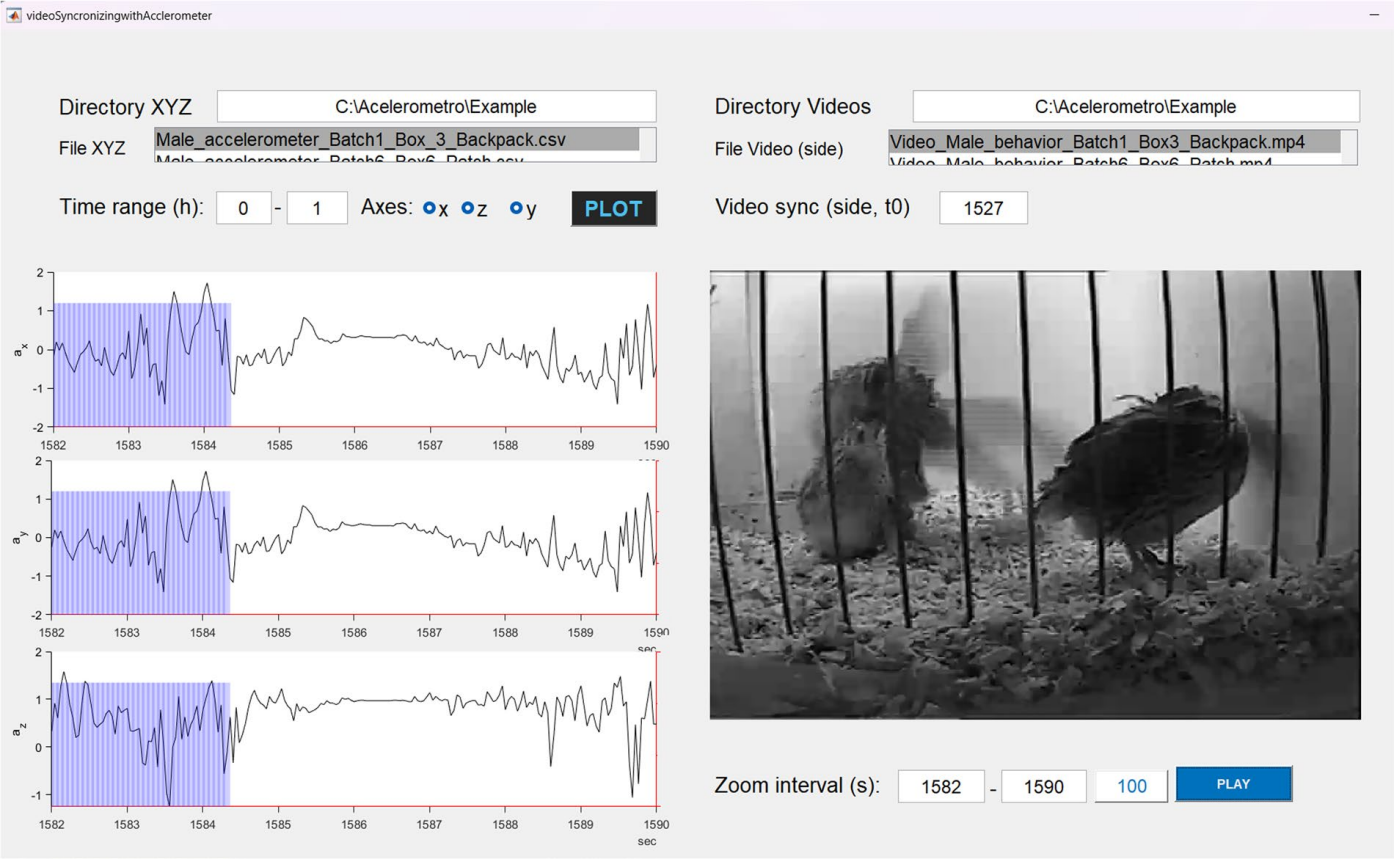
Screenshot of MATLAB app for visualizing simultaneously video recordings and acceleration vectors obtained from tri-axial accelerometer placed on the animal, synchronically. In the first two rows, users first specify the location and select the correct files to load the.csv file containing the acceleration data obtained from the accelerometer (following format specified in the section Data records) and the video files. In the third row users select the fragment of time in which he acceleration vector will be plotted and the synchronization parameter (t0_Acc_ in Table 8). By pressing plot, three axes of acceleration data are displayed (i.e. a_x_, a_y_ and a_z,_ black lines on plots). The zoom interval (bottom right) allows users to zoom in on a specific fragment of the acceleration data of interest, and then by pressing the play button users can observer the corresponding video fragment. Transparent vertical blue bars show the temporal reference between three axes of acceleration data and the progress of the video recording. The screen capture shows the exact time in which reproductive behavior was being performed (note the male mounted on female in right hand image).

One important characteristic of the dataset associated with the male-female interaction test is that three different sources of information can be compared: videos, observational data collected at high resolution from videos, as well as accelerometer data (see data usage section for details). Figure 5 shows an example MATLAB app that compares the time progression of the acceleration vector with the video recording. This app is publically available on Figshare^24^. Example code showing how behavioral data (eg. Shakes, Reproductive) can be visualized simultaneously with accelerometer data (eg. y-axis vector) in MATLAB is also provided on Figshare^25^.

## Data Records

The original video files^12^ are available in .mp4 format, and the time series^25^ as text files (.csv) on Figshare^12,25^. Data from each test and each individual is stored as a separate file as detailed in Table 7. Quails were identified by their Batch #, Box # and treatment. All information has been provided in the file name as “Test_Batch#_Box#_Treatment” (Table 7). Specifically, *Batch #* corresponds to the experimental group the animals were tested in, considering that within each batch a pair of females or male belonging to each treatment was tested. The *Box #* indicates the number of the home-box animals were housed in. The *Treatment* specifies whether the birds were of control, patch or backpack treatments. In tests where 2 females were tested simultaneously, the data of each is presented in separate columns.

Within each file, the column *Time (s)* is present, indicating the time expressed in seconds in which measurements were taken. Other columns with *Behavior* indicate the behavior being performed at a given time point recorded. Within each column a value of 1 indicates that the animal is performing the behavior at the given time point while a 0 represents that it is not performing the behavior.

In the case of male accelerometer recording files, a_x_, a_y_ and a_z_, provide acceleration recordings at each time point in the 3 axes (i.e., x, y and z). The first column provides the date and the second column the time-of-day accelerometer recordings were obtained. The following third column the x-axis, the fourth column the y-axis and the fifth column the z-axis of the acceleration vector.

Note that sampling rate varies depending on the test and whether behavioral data or accelerometer data is shown, as specified in the last column of Table 7, and evident in the column *Time (s)* of every file. Also, column with all zeros values indicates that the animals did not perform that behavior in the specific test.

## Technical Validation

All data analysis and technical validation was performed by one observer either using customized software developed in MATLAB or in ANY-maze. In both cases the observer was blinded regarding the prior history of the animals allocated in each group. In software keystrokes allow the observer to register manually behaviors from video recording. After training, a validation period to guarantee reproducibility was first performed where the observer analyzed a subset of three videos twice. It is important to note that no behaviors went undetected. Only slight differences (less than 1 sec) between the duration of behavioral events were observed in few cases when using the ANY-maze software.

## Usage Notes

In order to compare behavioral recordings to accelerometer recordings the offset between them needs to be estimated. Table 8 provides the required offset (t0) for each file obtained by detailed analysis of recordings (see Section Data Records for details of file names) and can be used to visualize the time point in seconds from the original video recordings^12^ (see example Fig. 5). For each file the offset is provided in seconds as t0_Beh_ for the behavior data and t0_Acc_ for the accelerometer data (Table 8).

**Table 8:** Estimated offset between behavioral and accelerometer files.

Male behavior file	Male accelerometer file	Offsets (s)
Male_behavior_Batch1_Box 3_Backpack	Male_accelerometer_ Batch1_Box 3_Backpack	t0_Beh_ = 1500.4
		t0_Acc_ = 1527
Male_behavior_ Batch2_Box 1_Backpack	Male_accelerometer_ Batch2_Box 1_Backpack	t0_Beh_ = 1186.2
		t0_Acc_ = 1150.5
Male_behavior_ Batch2_Box 2_Patch	Male_accelerometer_ Batch2_Box 2_Patch	t0_Beh_ = 1482.5
		t0_Acc_ = 1450
Male_behavior_ Batch5_Box 1_Patch	Male_accelerometer_ Batch5_Box 1_Patch	t0_Beh_ = 1262.75
		t0_Acc_ = 1252.68
Male_behavior_ Batch5_Box 2_Backpack	Male_accelerometer_ Batch5_Box 2_Backpack	t0_Beh_ = 950.5
		t0_Acc_ = 942.1
Male_behavior_ Batch6_Box 6_Patch	Male_accelerometer_ Batch6_Box 6_Patch	t0_Beh_ = 720.9
		t0_Acc_ = 712

As stated previously, the experimental protocol of the male-female interaction test includes a series of timed pauses and movements important for this synchronization. Due to the high speed of reproductive behavior, the values of the offsets were corroborated and fine-tuned by visually comparing behavioral time series obtained from video recording with acceleration vectors. Focus was placed on body shacks since they are events that predominantly last 70 milliseconds^10^, and are clearly evident as a high amplitude peak in the three axes of the acceleration vector. It also should be recalled that, as stated previously, behavioral recordings are provided at a frame-by-frame basis, hence a resolution of 1/15 s while accelerometer recordings were obtained at a higher resolution of 1/25 s.

## Data Availability

MATLAB code for recording behavioral data is publically available as an app on Figshare^11^. Interface allows researchers to customize sampling intervals, determine the number of animals analyzed simultaneously and synchronize both lateral and top video cameras for analysis. The output has a first column denoting the frame sample was taken and the following columns the behavior of each animal at the corresponding time point. The commercially available ANY-maze Video Tracking System software can be downloaded at www.anymaze.com. Example codes for (i) an app that shows synchronized acceleration vectors an video recordings^24^ and (ii) visualizing simultaneously Behavioral (eg. Shakes, Reproductive) and Accelerometer data (eg. y-axis vector)^25^, are provided on Figshare.
